# Ductal carcinoma in situ arising in tubular adenoma of the breast

**DOI:** 10.1007/s12282-012-0375-9

**Published:** 2012-06-15

**Authors:** Michiyo Saimura, Keisei Anan, Shoshu Mitsuyama, Minoru Ono, Satoshi Toyoshima

**Affiliations:** 1 Department of Surgery, Kitakyushu Municipal Medical Center, 2-1-1 Bashaku, Kokurakita-ku, Kitakyushu, Fukuoka 802-0077 Japan; 2 Department of Radiology, Kitakyushu Municipal Medical Center, 2-1-1 Bashaku, Kokurakita-ku, Kitakyushu, Fukuoka 802-0077 Japan; 3 Department of Pathology, Kitakyushu Municipal Medical Center, 2-1-1 Bashaku, Kokurakita-ku, Kitakyushu, Fukuoka 802-0077 Japan

**Keywords:** Tubular adenoma, Ductal carcinoma in situ, Breast carcinoma

## Abstract

We herein report an extremely rare case of ductal carcinoma in situ (DCIS) arising in tubular adenoma of the breast. A 33-year-old female first noticed a mass in her right breast when she was 15 years old. The tumor had not changed in size subjectively for 18 years. She finally visited the hospital one and a half years before this presentation for an examination of her breast mass. Ultrasonography (US) showed a circumscribed mass suggesting a benign tumor, and mammography (MMG) revealed the well-defined high-density mass with a focal region of microcalcification. It was suspected to be adenosis based on a core-needle biopsy (CNB). During the regular follow-up, the microcalcification in the mass increased. She was therefore referred to our hospital for further examination. US and MMG showed a well-demarcated mass with a focal microcalcified area. US-guided CNB diagnosed it as DCIS with tubular adenoma. The patient underwent tumorectomy. Histologically, the tumor was diagnosed to be DCIS in tubular adenoma with negative surgical margins.

## Introduction

Tubular adenoma is a rare benign tumor of the breast, which was first reported as a distinct entity called “pure adenoma” by Persaud et al. [1]. Then in 1976, Hertel et al. [2] histologically classified breast adenomas as follows: (1) true adenoma, including tubular adenoma, combined tubular and fibroadenoma, lactating adenoma and sweat gland tumors, (2) nipple adenoma, (3) fibroadenoma. Tubular adenoma of the breast is characterized histologically by a circumscribed mass consisting of prominent lobular proliferation and closely packed small ducts with minimal supporting stroma [3]. These uniformly sized ducts are lined by double layers of epithelium and myoepithelium. Due to the rare occurrence of tubular adenoma, malignant transformation of tubular adenoma and concurrence of tubular adenoma and carcinoma have been reported in only three cases. We herein report a case demonstrating ductal carcinoma in situ (DCIS) arising in tubular adenoma.

## Case report

A 33-year-old female first noticed a mass in her right breast when she was 15 years old. The tumor had not changed in size subjectively for 18 years. She finally visited a hospital one and a half years prior to this presentation to examine her breast mass. Ultrasonography (US) showed a circumscribed mass suggesting a benign tumor, and mammography (MMG) revealed a microcalcification in the mass. It was suspected to be adenosis based on a core-needle biopsy (CNB). During the regular follow-up, the microcalcification in the mass increased. She was therefore referred to our hospital for further examination.

**Fig. 1:**
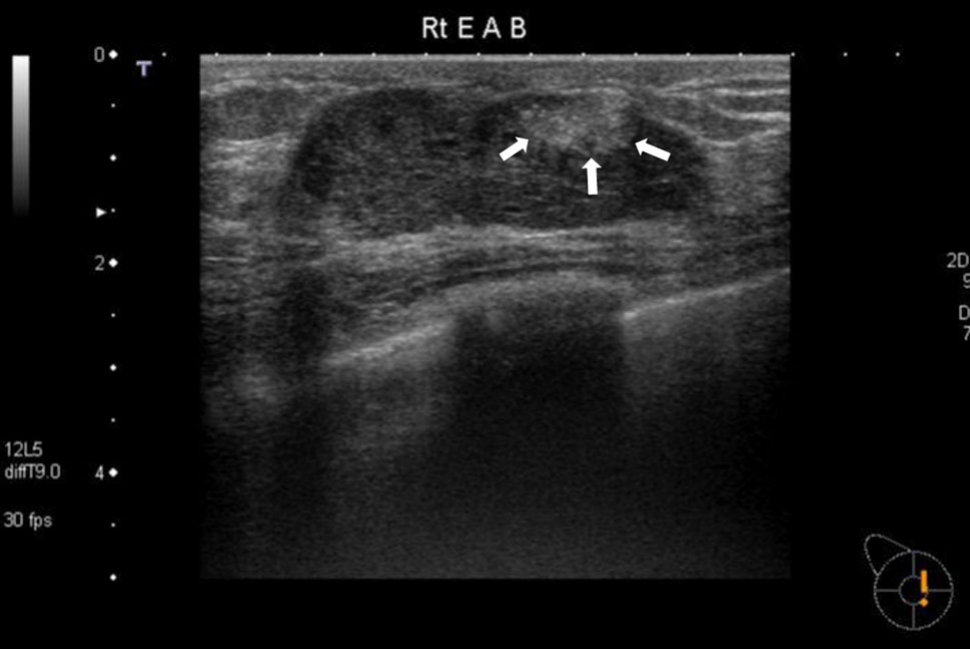
US revealed a well-demarcated hypoechoic mass 4.7 cm in diameter, and a focal hyperechoic area with an echogenic spot 1.2 cm in diameter within it (*arrow*)

**Fig. 2:**
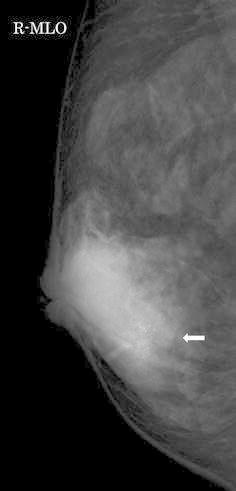
MMG showed a circumscribed high-density mass with a grouped punctated or amorphous microcalcification in the mass, whose area was about the same as the hyperechoic area on US (*arrow*)

A physical examination revealed a discrete and freely movable mass 5 cm in diameter beneath the nipple in her right breast. There was no nipple discharge. She had no history of oral contraceptive use or pregnancy. US revealed a well-demarcated hypoechoic mass 4.7 cm in diameter, and a focal hyperechoic area with an echogenic spot 1.2 cm in diameter within it (Fig. 1). MMG showed a circumscribed high-density mass with a grouped punctated or amorphous microcalcification in the mass, whose area was about the same as the focal hyperechoic area on US (Fig. 2). The calcified area was suggested to be malignancy, although the whole mass was thought to be benign. Computed tomography (CT) revealed a well-defined enhanced mass in the right breast. Dynamic contrast-enhanced magnetic resonance imaging (MRI) disclosed a circumscribed enhanced mass with a rapid-plateau pattern. Both CT and MRI could not identify the cancerous lesion within the tumor. US-guided CNB from the hyperechoic area diagnosed it to be DCIS in tubular adenoma.

**Fig. 3:**
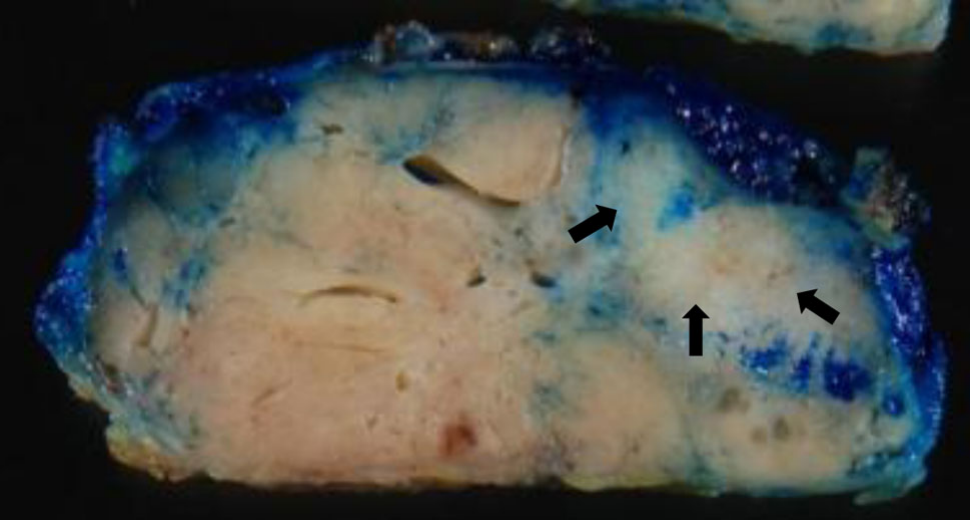
The tumor measured 4.7 × 4.0 × 2.2 cm in the greatest dimensions, and the cut surface showed a white part within a yellowish nodule, which was much the same as the hyperechoic area on US and the calcified area on MMG (*arrow*)

**Fig. 4:**
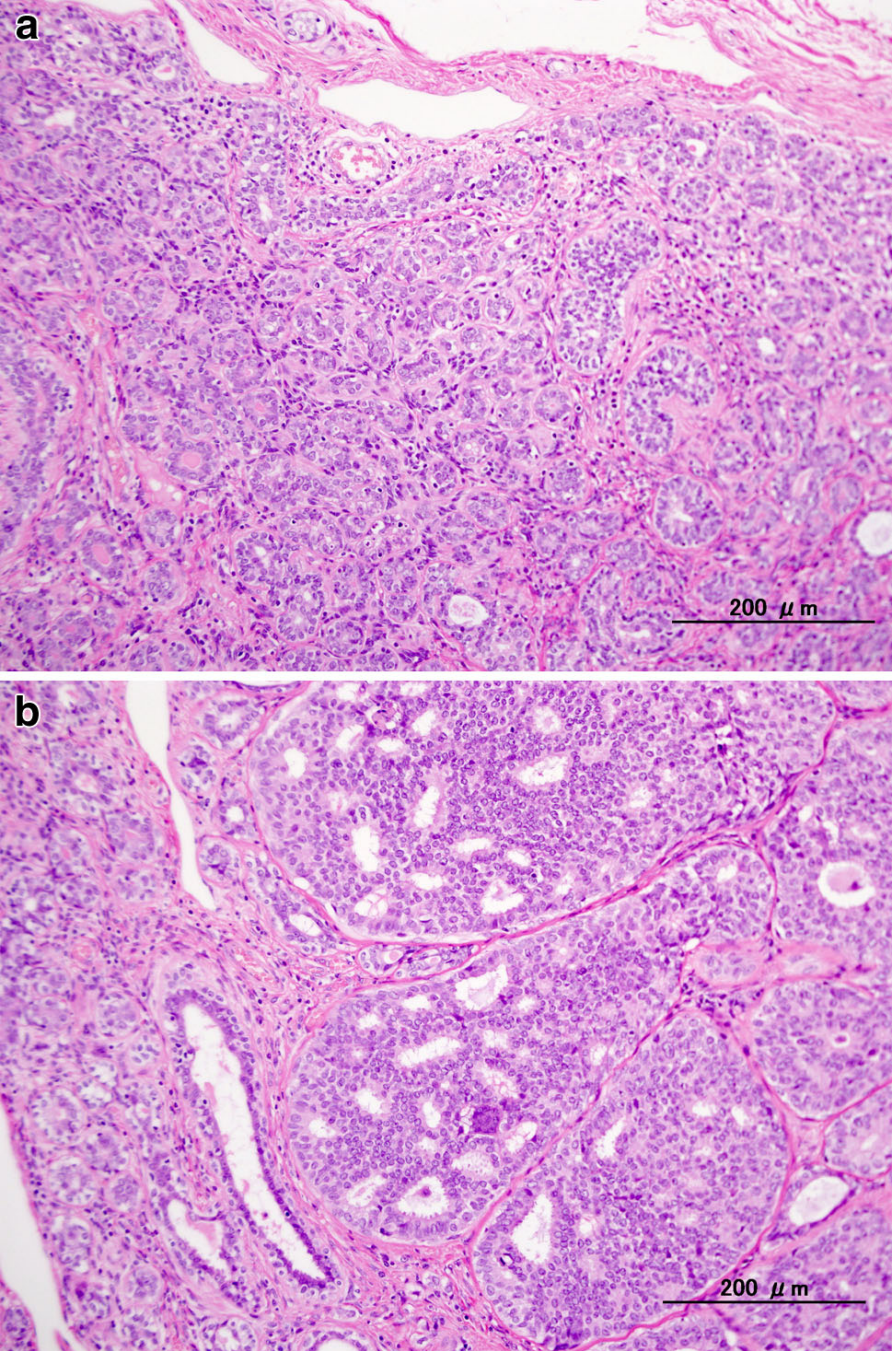
The tumor comprised two parts, with an indistinct border between them. **a** The main part of the tumor showed proliferation of uniform small ducts that were composed of double layers of epithelial cells and myoepithelial cells with a small amount of stroma. It was diagnosed to be tubular adenoma. **b** The other part consisted of neoplastic epithelial proliferation, in which microlumens were formed containing cellular debris and calcification that was detected on MMG. The microlumens were surrounded by homogeneous low-grade cuboidal to low columnar cells. It was diagnosed to be intraductal carcinoma

Because the carcinoma part was suggested to be surrounded by a benign tumor, we performed tumorectomy. The tumor measured 4.7 × 4.0 × 2.2 cm in the greatest dimensions, and the cut surface showed a white part within a yellowish nodule, which was much the same as the hyperechoic area on US and the calcified area on MMG (Fig. 3). Histologically, the tumor comprised two parts, with an indistinct border between them. The main part showed proliferation of uniform small ducts that were composed of double layers of epithelial cells and myoepithelial cells with a small amount of stroma (Fig. 4a). These tumor cells had round to oval nuclei with inconspicuous nucleoli, and lacked cytological atypia. Based on these findings by hematoxylin and eosin stain, it was diagnosed to be tubular adenoma. The MIB-1 index of epithelial cells was 13.9 %. The other part consisted of neoplastic epithelial proliferation with solid and cribriform patterns, in which the microlumens were formed containing cellular debris and granular or psammomatous calcification that was detected on MMG. There was no comedo necrosis. The microlumens were surrounded by homogeneous cuboidal to low columnar cells of low nuclear grade. This part was diagnosed to be intraductal carcinoma. The histological transition between DCIS and tubular adenoma was not determined, but DCIS existed within the tubular adenoma and had spread into it (Fig. 4b). The surgical margin was negative. The histological boundary between this tubular adenoma and the surrounding breast tissue was clear, and there was no cancerous tissue around it. The patient underwent no further treatment after surgery.

## Discussion

This is the fourth report of carcinoma arising in tubular adenoma of the breast and the first one of DCIS. The carcinoma component was suspected due to the increasing microcalcification first detected on MMG, and it was histologically diagnosed to be DCIS with tubular adenoma based on a US-guided CNB from the hyperechoic area in the demarcated mass. The patient underwent tumorectomy.

Tubular adenoma is a rare benign tumor of the breast, which is defined according to the World Health Organization classification as “benign, usually round, nodules formed by a compact proliferation of tubular structures composed of typical epithelial and myoepithelial cell layers” [4]. Tubular adenoma should be differentiated from adenosis tumors, which are characterized by focal hyperproliferation of fibrocystic mammary glands [5]. In reference to Nielsen’s criteria for the differential diagnosis between tubular adenoma and adenosis tumors, the present tumor had a homogeneous configuration, uniform growth pattern, round glands and the absence of microcysts, apocrine metaplasia, elastosis, glomeruloid structures, and microcalcification. From these findings, the benign part of the tumor was diagnosed as tubular adenoma. The histological differentiation of tubular adenoma from fibroadenoma may cause diagnostic difficulties in cases of tubular adenoma with a relatively abundant stromal element, or fibroadenoma with significant proliferation of small ducts. Ductal adenoma is distinguished from tubular adenoma by the benign intraductal proliferation, which is suggested to be the sclerotic evolution of an intraductal papilloma. In the present case, the benign part of the tumor showed proliferation of uniform small ducts that were composed of double layers of epithelial cells and myoepithelial cells with scarce stroma. There was no myoepithelial proliferation typical of adenomyoepithelioma, and the epithelial cells showed no apocrine metaplasia characteristic of apocrine adenoma.

Due to the rare occurrence of tubular adenoma, malignant transformation of tubular adenoma and concurrence of tubular adenoma and carcinoma have rarely been reported. To the best of our knowledge, only three cases of carcinoma arising in tubular adenoma have been reported previously. In 1954, Hill and Miller [6] described the first case of a 34-year-old female with breast nodule showing liver metastasis. The histological diagnosis by biopsy was adenoma at first, but close reexamination revealed the presence of minute areas of invasive carcinoma within the tubular adenoma, and the metastatic foci were histologically consistent with metastasis of the breast carcinoma. The second case was the adenocarcinoma of the breast arising in a preexisting adenoma [7]. The third case was the colocalization of tubular adenoma and an invasive ductal carcinoma, and was histologically speculated to be a collision of a separate tubular adenoma and invasive ductal carcinoma [8]. In the present case, the tubular adenoma had appeared 18 years prior and hardly changed in size. It was unclear when the DCIS arose, but it was highly suspected that it was the subsequent carcinoma in the preexisting tubular adenoma. Although the histological transition between DCIS and tubular adenoma was not determined, DCIS was found to be completely surrounded by the tubular adenoma and it had also spread within it. DCIS therefore appeared to have arisen within the tubular adenoma.

In the previous three cases, the findings on MMG were available in two cases. In one case, MMG showed a very sharply demarcated mass without calcification except at the margin where only a slight loss in the definition of the lesion was observed [7]. In the other case, MMG revealed a distinct tumor comprising two parts: a well-demarcated highly dense tumor surrounded by an irregular lesion with radiodensity [8]. No microcalcification was identified. In the present case, the microcalcification on MMG suggested a coexisting malignancy in the benign tumor. The diagnosis of coexisting carcinoma was obtained by biopsy in two cases [6, 7] and by cytological fine-needle aspiration in one case [8]. Under a diagnosis of malignancy, two patients underwent operation; one received mastectomy with axillary node dissection [7], and the other underwent partial excision with axillary node dissection [8]. The patient who also had liver metastasis did not undergo surgical treatment [6]. In the present case, the carcinoma component could be diagnosed by CNB preoperatively. Because it was diagnosed as DCIS on CNB and suspected to be completely surrounded by the benign lesion on images, we performed only tumorectomy. The excised tumor was well demarcated, and the surgical margin was negative. The precise preoperative diagnosis thus made it possible to perform minimally invasive treatment.

## Conclusions

Although the development of carcinoma is extremely rare in preexisting tubular adenoma, which is generally considered not to impart an increased risk of carcinoma, it is nevertheless important to be aware of the possibility of the coexistence of a benign and malignant lesion. When malignant findings appear on images, such as microcalcification on MMG in the present case, an appropriate approach for the diagnosis and definitive treatment should therefore be applied.
